# Outpatient hysteroscopy

**Published:** 2018-09

**Authors:** R Campo, F Santangelo, S Gordts, C Di Cesare, H Van Kerrebroeck, MC De Angelis, A Di Spiezio Sardo

**Affiliations:** European Academy of Gynaecological Surgery, Diestsevest, 43, 3000 Leuven Belgium.; Department of Obstetrics and Gynecology, ZOL Hospitals, Schiepse Bos 6, 3600 Genk, Belgium; Life Expert Centre, Schipvaart straat 4, 3000 Leuven Belgium; Unit of Obstetrics and Gynecology, University of Federico II, Naples, Italy, 80131; Catholic University of Sacred Heart, Department of Obstetrics and Gynecology , Fondazione Policlinico Universitario Agostino Gemelli, Rome, Italy

**Keywords:** hysteroscopy, outpatient, office, see and treat, one stop, uterus

## Abstract

Modern hysteroscopy represents a copernical revolution for the diagnosis and treatment of uterine pathology. Traditionally hysteroscopy was performed in a conventional operation room under general anaesthesia (in-patient hysteroscopy). Recent advances in technology and techniques made hysteroscopy less painful and invasive allowing it to be performed in an ambulatory setting (outpatient hysteroscopy).

The so called “see & treat hysteroscopy”, has reduced the distinction between diagnostic and operative procedure, thus, introducing the concept of a single procedure in which the operative part is perfectly integrated within the diagnostic work-up.

The “digital hysteroscopic clinic” (DHC) on the other hand combines ultrasound with hysteroscopy, ideal for a one stop diagnostic procedure and surgical approach, outlasting laparoscopy with ultrasound, for increased surgical performance in outpatient settings. The aim of this paper is to describe the “state of the art” in an outpatient hysteroscopy setting.

## Introduction

Technical and technological advancements in gynecology have continuosly revolutioned our everyday life. This is particularly true in the field of gynecological practice where the introduction and development of endoscopic and ultrasound technology has lead to exciting results. More specifically, in the world of endoscopy, hysteroscopy (from the Greek term hysteros: uterus and scopy: to look) is a real copernical revolution for modern gynecology. The application of this technique (hysteroscopy), for a first time, helped to “elucidate” an obscure, unexpolored and isolated region of the human body left a part since the middle of the ninteenth century, the uterus. Currently, hysteroscopy can be considered, in every respect, the gold standard for examination of the uterine cavity permitting to bypass the significant limitations of dilatation and curettage (D&C).

Traditionally, diagnosis and treatment of intrauterine diseases was based, almost exclusively, on curettage. Some scientific societies continue to promote the diagnostic and therapeutic role of D&C. However, D&C, typically performed under general anesthesia, is characterized by a poor ability to discriminate focal lesions [within the uterine cavity] which result in an extremely high percentage of false negatives and thus a low therapeutic profile. Additionally, in the recent years several studies have put forward the major limits of this technique: blind dilatation. Importantly, the latter entails a high risk of perforation.

Since the beginning of the 80’s, hysteroscopy has become a powerful diagnostic tool to visualize the cervical canal and the uterine cavity, by performing the so-called ‘traditional technique’. This approach involves the use of a speculum and cervical clamp, allowing for the examination of the cervix and cavity, mainly by CO 2 distension. Since preparatory cervical dilatation is mostly mandatory, trauma and bleeding provoked by the diameter of the instrument, caused hysteroscopy to be more difficult and time consuming.

Due to the large diameter of hysteroscopes and the use of CO 2 as distention medium (significant more painfull than watery solutions) the exam could mostly not be performed in an outpatient setting, but generally required general anesthesia and one day or overnight hospitalisation (“in-patient hysteroscopy”).

In the early 90’s, the use of low viscosity fluid and technological advances permitted the reduction of the diameter of hysteroscopes. This turned-over, made hysteroscopy less painful and minimally invasive, hence, drastically reducing the number of procedures performed in operating rooms. In consequence, the number of ambulatory procedures (“outpatient hysteroscopy”) was increased. Yet, this may also be attributed to the fact that outpatient hysteroscopy has the inherent benefit of obviating the need for general anesthesia and dilatation of the cervical canal.

Stefano Bettocchi, who contributed significantly to the development and spread of ambulatory hysteroscopy, introduced the atraumatic sight controlled vaginoscopy (or no touch technique) in which the scope is introduced in the vagina using a watery solution to inspect the external cervical opening, cervical canal, internal ostium and cavity without the use of a speculum or a tenaculum.

For this, a hysteroscope equipped with an operating channel for instruments up to 5 Fr., including a novel sheath with a diameter of only 5 mm and an ovale shaped cross-section for increased ergonomy, and thus, comfortability of the procedure, upon contact with the internal uterine ostium (IUO) was developed.

Later, Campo developed the Campo Trophyscope ® , a compact 30° rigid 2.9 mm scope with a specially designed tip for atraumatic passage through the cervical canal. This instrument also included an innovative feature permitting it to be loaded with accessory sheaths in active and passive positions. The instrument allows for enlarged visuals during the procedure, the sampling of endometrium but also surgical actions for anatomopathological examination of the endo- and myometrium, without removing the instrument (Campo et al., 2014; El-Thoukhy et al., 2016).

Recently, in a prospective randomized controlled trial (the Trophy study), 320 hysteroscopies in patients with failed IVF performed in 8 different centres resulted in no complications and no acces failure, clearly demonstrating the high succes rate when using this new small bored optical system (El-Thoukhy et al., 2016). As a result, modern technological advancements have brought diagnostic hysteroscopy to a mainstay in today’s gynaecological practice. Ambulatory visualization of the uterine cavity has obvious major benefits for the patient and diagnostic procedures. Combining ultrasound and hysteroscopy into a single environmet including highly technological digital examination rooms, allows to reduce the time of ambulatory surgery while upholding accessibility for all diagnostics and also up to 90 % of all hysteroscopic operative interventions, thus increasing patient satisfaction and safety (Campo et al., 2014).

## Setting

Today, outpatient hysteroscopy should be carried out in a properly equipped ambulatory examination room counting with sanitary facilities and a dressing room. First and foremost, the patient must be welcomed in a familiar and relaxing environment ensuring a comfortable gynecological session. A qualified nurse should oversee the room in which the procedure will take place ascerting that all the equipment and tools necessary are available and inform the patient about the investigations to be performed. Indeed, it has been observed that women anxious before the procedure, usually experience greater discomfort during the intervention. Therefore, by reducing patient’s anxiety, positive results and maximum satisfaction can be expected.

**Figure 1:**
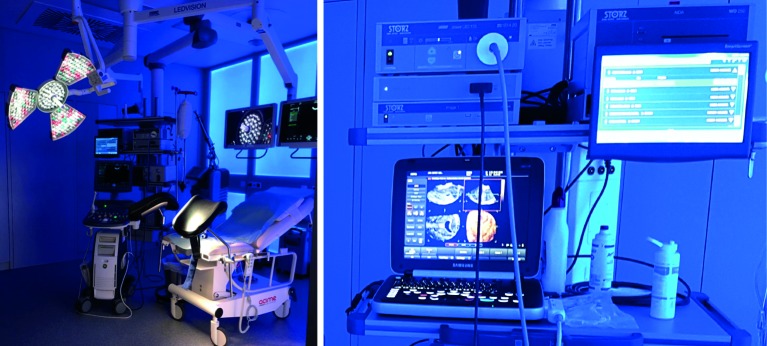
— DHC is equiped with a special wall delivering illumination with a desired color. A high definition camera system and a 3D ultrasound are connected to a digital patient report system.

The Digital Hysteroscopic Clinic (DHC) is equiped with a special wall delivering illumination with the desired color improving patient’s comfort and increasing the contrast over the endoscopic monitors (Figure 1).

During hysteroscopy it is therefore recommended to provide the patient with emotional support (“vocal anesthesia” - to reassure and involve her), for instance, by inviting her to look at the (additional) monitor while giving her explanations of what is seen (e.g anatomical structures, abnormalities) in order to prevent feelings of exclusion and/or neglect.

## Instruments

As mentioned above, for several decades, rigid hysteroscopes had a diameter of over 5 mm. In consequence, its use required cervical dilatation and anesthesia causing discomfort and contrariety. To solve this issue, in the last ten years, the industry has develloped rigid 30° optics with a diameter of less than 3 mm for diagnostic purposes.

**Figure 2:**
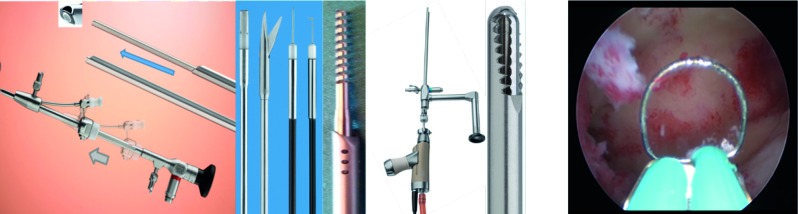
— Hysteroscopic instruments have similar total diameter so that interchange is possible . a. Campo Trophyscope ® b. 5 French instruments including spirotome inserted in the continuous flow examination sheath c. 19 Fr. Intrauterine BIGATTI Shaver (IBS ® ) d. 15 Fr. Office Resectoscope

In this line, the Campo Trophyscope ® has the advantage to start an examination with a compact 2.9 mm atraumatic rigid hysteroscope and accessory sheet in a passive position, not interfering with the diagnostic visualisation. In case of pathology, an accessory sheet is pushed forward for a visually controled dilatation, thus reducing the risk of discomfort, perforation or inadverted lesion of the fragile endometrium. Furthermore, this instrument can also be equipped with a continuous-flow sheet and a semirigid 5 Fr. intrument to ease any surgical action. Also, one can remove the hysteroscope and use the accessory sheet to introduce the Trophy curette or an endomyometrial sampler (Figure 2). As a result, anatomo pathological examinations of endometrium and myometrium and even minor interventions like polyp or myoma resection are possible whithout the need of a speculum in a one stop procedure.

It is to note that in case of in-office procedures, the compatibility of this instrument with a high level biodegradable desinfection agent like Tristel Fuse ® offers the possibility to reuse the instrument within a few minutes making the procedure accessible for every office gynecologist, ambulatory unit or IVF-center.

Likewise, flexible fiber-optic hysteroscopes have improved significantly in recent years. However, their use remains limited, especially for vaginoscopy, where passage of the cervical canal frequently needs a counter traction using a cervical clamp rendering this approach difficult. Additionally, its high cost and limited surgical possibilities, mainly due to labour-intensif cleaning and sterilization procedures, make of this approach a secondary option for outpatient hysteroscopy.

In outpatient hysteroscopy, saline solution and CO 2 are the most common media for intracavitary distention. Although CO 2 is generally well tolerated, uterine distension with saline solution is preferable. Distension with fluid is a cost-effective approach associated with less patient discomfort and clearer hysteroscopic vision in the occurrence of intra-uterine bleeding.

For the vaginoscopic approach liquid distension medium is required. Indeed, during surgical manoeuvres using electrical energy, watery solutions are mandatory. Distention with a watery medium can be administered at atmospheric pressure, by means of a pressure cuff or employing an automated microprocessor with flow and pressure control. The newest generation of fluid pumps has contributed to the improvement of patient comfort by measuring and controling the intrauterine pressure. Former generations of pump systems (or cuff pressure) only delivered a predefined pressure resulting in a large variety of intra urterine pressures.

In the vaginoscopic approach, passage through the intenal cervical os and the intra uterine pressure created by the distention medium and in case of CO 2 the irritation of the peritoneum when gas is passing the fallopian tubes, can provoke pain.

Recently, it has been recommended to enlarge the endoscopic examination room with a simple but high end three dimensional (3D) ultrasound desktop implemented in the endoscopic tower. 3D ultrasound has received increasing value for the diagnosis of uterine malformations and adenomyosis (Molinas and Campo, 2006; Gordts et al., 2018).

**Figure 3:**
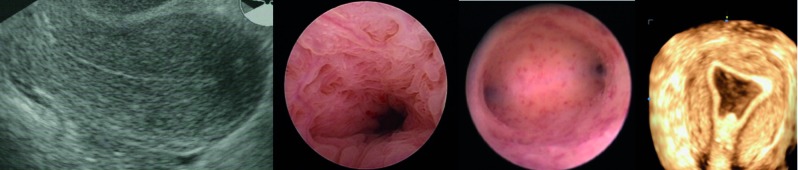
— The one stop diagnosic exam includes transvaginal ultrasound (2 or 3 D) followed by hysteroscopy and contrast sonography of the uterine cavity.

A one stop uterine diagnosis includes a transvaginal ultrasound followed by a fluid mini hysteroscopy using the vaginoscopic technique. Interestingly, this allows to perform a vaginal ultrasound using the hysteroscopy fluid as contrast to evalutate the uterus in its entirety (Figure 3).

## Outpatient diagnostic hysteroscopy

The improvements in techniques and endoscopic instrumentation including the implementation of ultrasound have allowed physicians to achieve more reliable results regarding the diagnosis of pathologies such as polyps, myoma, synechiae, placental remnants, intrauterine device (IUD) malposition, adenomyosis and congenital malformations (Campo et al., 2014; Gordts et al., 2018).

On the contrary, problems for endoscopists appear when performing endometrial or myometrial tissue sampling to diagnose forms of hyperplasia, explore subtle lesions, and diagnose adenomyosis or tissue sampling of a myoma to exclude malignancy.

Compared to traditional methods such as D&C, hysteroscopy offers multiple advantages. It enables to visualize macroscopic or focal abnormalities suggestive of endometrial hyperplasia inside the uterine cavity, and to perform targeted hysteroscopic biopsy (THB) withdrawal under visual control. However, due to the lack of established hysteroscopic criteria for the diagnosis and classification of endometrial hyperplasia and its overlapping pattern with the normal late secretory endometrium, mainly in premenopausal women, doubts about the reliability of endoscopic diagnosis (using “only the visualization of the uterine cavity”) subsist. Yet, considering the typical malignant evolution of endometrial hyperplasia, early hysteroscopic diagnosis of this condition may represent an advantage for the gynecologist, if associated with THB.

Endometrial sampling devices, such as the Vabra, Pipelle or Novak, face the same problems due to their “blind” application. In case of generalised lesion, these devices can be useful if applied in combination with a pre- and postbiospy hysteroscopic control.

The Campo Trophyscope ® hysteroscope provides the possibility to use the outer sheet as a guide to insert an endometrial sampler or an endometrial – myometrial biopsy probe called the spirotome (Figure 2).

The spirotome consists of a cockscrew and cutting sheet which permits to collect a biopsy of up to 2 cm length under ultrasound guidance. This technique claims to collect tissue with a minimal risk of cell spreading and bleeding. In practice, after removing the spirotome the Trophy optic can be reinserted to inspect the area where the biopsy was collected (Janssens et al., 2006).

Likewise, another common technique, the so-called “grasp” biopsy, must be taken into account. In this technique, biopsy forceps are placed, jaws open, against the endometrium, then the forceps are pushed into and along the tissue for half or one cm. Finally, the two jaws are closed and the whole hysteroscope is pulled out of the uterine cavity, without pulling the tip of the instrument back into the channel. Under this procedure, THB [to confirm the “visual” diagnosis] can be performed regularly by a trained physician.

## Outpatient operative hysteroscopy

The “see & treat hysteroscopy” or outpatient operative hysteroscopy, a new outlook in hysteroscopic practice, was developed in the late 90’s. This practice introduced the concept of a single procedure perfectly integrating the operative part in the diagnostic work-up. This was made possible thanks to the advent of small diameter scopes (with a 5 Fr. operative channel).

The use of miniaturized mechanical instruments (without any anesthesia) together with the use of small diameter scopes with working channels and continuous flow systems, persist to be the only way to apply “see & treat” hysteroscopy in an outpatient setting. In 1997, the introduction of a versatile electrosurgical bipolar system for hysteroscopy, the Versapoint (Gynecare, Ethicon, NJ, USA), represented a turning point in in-office operative hysteroscopy. Thanks to the use of 5 Fr. bipolar electrodes, the number of pathologies treated in office operative hysteroscopy increased greatly, thus, reducing the use of resectoscopes and operating rooms to specific cases. A major advantage of these new instruments is the use of bipolar (rather than monopolar) energy. This allows for the use of a saline solution rather than non-ionic distension media (e.g. glycine, sorbitol, mannitol), plus reducing the energy spread through the tissue during resection. In 2000, Lindheim et al. (2000) demonstrated the potentials of operative hysteroscopy in an office setting, showing that in a group of self-selected patients, operative procedures were successfully performed in 97% of cases with mechanical instruments and bipolar electrodes.

In 2002, Bettocchi et al. reviewed the benefits of this new 5 Fr bipolar electrosurgical equipment. In the treatment of large benign intrauterine pathologies the combination of new generation (smaller diameter) hysteroscopes and the Versapoint system eased the practice of in-office hysteroscopy for the collection of endometrial biopsies, uterine adhesiolysis, as well as the treatment of polyps and myomas smaller than 1.5 cm, with excellent patient tolerance. Recently, Di Spiezio Sardo et al. (2010) showed that even other uncommon gynaecological pathologic conditions could be treated safely and effectively in an office operative hysteroscopy setting.

## Digital hysteroscopic clinic (DHC)

A DHC setting is a new concept in precision medicine. It includes the combinination of in-office and ambulatory surgical care and upgraded imaging technology for accurate uterine diagnosis and treatment. 3D ultrasound imagery is integrated in the endoscopic tower and the combination of ultrasound (US) and hysteroscopy and eventually magnetic resonance imaging (MRI) provides a total new concept of uterine diagnostic and therapeutic approach. The DHC clinic is characterized by a patient friendly organization combined with a major procedural capacity of up to 50 procedures per room.

This totally new concept, has been studied at the Genk ZOL hospital in Belgium for the last 10 years. In our experience, out of 2777 consecutive hysteroscopic surgical procedures, 86 % (2388) of these interventions were performed in an ambulatory setting with no registered complications when using miniaturized instruments (Table I). In consequence, miniaturization and interchangeability of hysteroscopic instruments can be associated with increased patient compliance and reduced complication rate (Campo et al., 1999; 2005; 2014).

**Table I:** ZOL Genk, Belgium ambulatory hysteroscopic surgeries in comparision to conventional operating room surgery.

Procedure	N total	% all surgeries	N Outpatient	N In patient	% Outpatient
Ashermann	114	4.1	114	0	100
Uterusplasty/septum	454	16.3	436	18	96
Others Replacement IUD Adenomyosis, sectio niche	292	10.5	280	12	96
Placental remnants	211	7.6	187	24	89
Polyp resection	902	32.5	798	104	88
Endometrium resection	503	18.1	389	114	77
Myomectomy	301	10.8	179	122	59
Total	2777	100	2383	394	86

## Indications and limits for outpatient operative hysteroscopy

Currently, appropriate equipment, proper training and knowledge are sufficient to practice safe in-office operative hysteroscopy. In the present section, we review ambulatory setting treatments of common endouterine diseases.

With the introduction of DHC nearly 90 % of all hysteroscopic surgeries can be performed in an outpatient setting. However, it is to note that patients with an ASA score > 2 and large pathologies, where surgery will exceed 30 min, and the requirement of large diameter instruments, are contraindications for ambulatory (in-office) surgery. In the practice, the surgery should be performed within one hour and post-operative observation of the patient should not require a high tech recovery infrastructure. This is possible thanks to the advantages of minimal anesthesiological interventions, efficient and fast patient turn over, centralisation of high tech equipment and reduced costs related to dedicated personnel and operating room usage, all included in the DHC setting. Thus, the efficacy of conventional operating room surgery is improved by the avoidance of minor procedures, non-applicable to a DHC setting. Today, the standard set up in DHC includes a 3D desktop ultrasound machine (with abdominal and vaginal probe), a Campo Trophyscope ® hysteroscope 2 and a 2,9 mm 30° fore-oblique lens system, the 15 Fr. bipolar office resectoscope and the 19 Fr. Intrauterine BIGATTI Shaver (IBS ® ). For fluid administration the Hysteromat E.A.S.I system is recommended (Bigatti, 2011; Bigatti et al., 2014) (Figure 2).

## Conclusion

The availability of a HPV vaccine in a country is not a guarantee of success, not even when it is offered free of charge. The situation in both communities in Belgium illustrates the role different factors may play in different ways: differences in awareness, spill over effects of the media in neighbouring country, different penetration of vaccination by SHS in each part of the country. Understanding the role of these factors and tailoring the implementation of the HPV programme to the needs of each community is key, in addition to efforts to raise knowledge and awareness among the respective health care providers and parents in each part of the country.

Polypectomy: endometrial and cervical small polyps (< 0.5 cm), should be removed using a 5 Fr. mechanical instrument such as a sharp scissor and/or crocodile forceps (Figure 2).

Larger polyps up to 2 cm can be removed using bipolar energy. For this, a 15 Fr. office resectoscope or the Versapoint Twizzle electrode are recommend. Depending on the internal cervical ostium size slicing prior to extraction should be resolved. Slicing is done with the 15 Fr. office resectoscope or with the Twizzle by cutting the polyp from its free edge to its base and sliced into two to three fragments, large enough, to be pulled-out of the uterine cavity using 5 Fr. grasping forceps with teeths.

Very large polyps or general polyposis are preferentially treated with a shaving system. It has been demonstrated that with new pump systems, shaving instruments are a safe and easy-to-learn option allowing for the fast removal of soft tissue (Di Spiezio Sardo and Nappi, 2014).

Myomectomy: In cases of myomas, ambulatory surgery is limited by the size, position and hardness of the myoma. In Type 0 myoma a technique similar to polypectomy is applied, with minor differences. Due to the higher density tissue density, myomas must first be divided into two half-spheres and then each of these sliced (as described for polyps).

For Type 1, 2 and 3 myomas presenting an important intramural proportion, first, a gentle separation of the myoma from the capsule using mechanical instruments (scissors, grasping forceps) and the bipolar needle to pin point coagulation of the vessels is required. This step avoids myometrial stimulation or damage of the surrounding healthy myometrium, plus, coagulates important afferent and efferent vessels prior to a shaping procedure. After, to slice the myoma, either a shaver, a 15 Fr. office resectoscope or a bipolar needle like the Versapoint Twizzle electrode, can be used.

Recently, a new ambulatory surgical technique to prepare large (> 1,5 cm) submucous myomas with partially intramural development (G1 and G2) in an outpatient setting with miniaturized Office Hysteroscopes (Office Preparation of Partially Intramural Myomas: OPPIuM) was put to practice. This technique facilitates the subsequent resectoscopic removal under general anaesthesia (Cicinelli et al., 2016). This technique consists in the incision of the endometrial mucosa and of the pseudo-capsule covering the myoma allowing to push the myoma into the uterine cavity by the myometrial fibers. Currently, in an ambulatory setting we limit treatment of myomas type 1-3 to a maximum size of 2 cm. To note, mild sedation improves the possibilities to remove myomas in an ambulatory setting, nevertheless, myomectomy remains the most frequent intervention which can not be transferred to the outpatient or ambulatory operating room.

Furthermore, when dealing with moymas > 2 cm, with concomitant pathology or in case of multiple myomas, it is reasonable to plan an in-patient procedure using a conventional resectoscope. Resectoscopic myomectomy for G0 and G1 myomas is not a complex procedure. On the contrary, it is a difficult procedure for G2 myomas, associated with a significant risk of complication. Indeed, the larger is the myoma and its intramural development, the more likely is the need for splitting the procedure into different surgical steps. (Di Spiezio Sardo and Nappi, 2014)

Acquired and congenital uterine cavity deformations: Asherman’s syndrom, dysmorphic uterus class 1 and septated uterus class 2 are the most accessible surgeries for an ambulatory approach. Although Asherman’s syndrom is the most difficult surgery, the use of small instruments and ultrasound is mandatory.

Both, dysmorphic and septate uterus anomalies are very common in the infertile patients. Evidence from their association with implantation failure and recurrent miscarriage is well described in the literature (Homer et al., 2000; Rackow et al., 2007; Saravelos et al., 2008, Di Spiezio Sardo et al., 2015, Ferro 2019). In these patients, metroplasty using microscissors and bipolar electrodes have clearly demonstrated to increase reproductive outcomes.

Before the onset of a hysteroscopic approach, treatment of the uterine septum was performed by an abdominal surgical approach. Nowadays, this defect is easily treatable by incising the septum with a hysteroscope. In this regard, the possibilities of performing metroplasty in an outpatient setting using micro scissors or electrodes, without any prior laparoscopic information, is an advantage.

In 2016 Di Spiezio Sardo et al. showed that the use of 3D-TVS, in the case of uterine septa, provides repeatable and objective measurements of the septimentation. This, combined to intraoperative objective data from graduate intrauterine palpator data, allowed achieving complete removal (fundal notch &10 mm) of the uterine septum in one outpatient surgical step. In this manner, the risk of residual septum, mainly due to the surgeon’s subjective assessment of the resection and lack of standardized pre- and post-operative evaluation of the uterine cavity was eliminted.

A dysmorphic uterus is a mullerian anomaly which, if untreated, is associated with poor reproductive outcomes in patients needing in vitro fertilization treatment. (Ferro, 2019) This malformation can be treated in an outpatient setting (with conscious sedation), thus avoiding invasive traumatic techniques. In surgery, longitudinal lateral incisions are performed on the fibro-muscular constriction rings in the isthmic area with a 5 Fr. bipolar electrode or scissors. With this, this technique improves volume and morphology of the uterine cavity (Di Spiezio Sardo and Nappi, 2014, Ferro, 2019).

## Recommendations

All gynecologists should provide special outpatient hysteroscopy services to provide diagnosis and treatment to women with abnormal uterine bleeding, infertility and intrauterine abnormalities. This procedure is called “outpatient hysteroscopy”. So far, its application has demonstrated clinical significance and economic benefits (Grade II evidence, Grade A recommendation).

However, outpatient hysteroscopy practitioners must have the proper skills and expertise, mandatory to perform hysteroscopy (Level VI, Recommended Intensity A). Patient information must be provided prior to surgery and informed consent must be signed by the patient (Level VI, Recommended Intensity A). Diagnostic hysteroscopy and, if necessary, biopsy or even surgery should be performed in a well-equipped, well-staffed informal operating room to ensure patient safety and privacy (level of evidence II, recommended intensity B) (Practical guideline SEGI, 2013).

## Conclusions

Although operative hysteroscopy has progressively been accepted for the treatment of intrauterine pathologies, diagnostic hysteroscopy and ambulatory surgery, it is still not widely used in routine. Nevertheless, compared to most urologists who utilize office cystoscopy to evaluate bladder pathology, it is estimated that less than 20% of gynecologists utilize office hysteroscopy to evaluate uterine pathology.

The use of watery distention medium and atraumatic sight control vagino- cervico insertion of the scope has been reported as one of the keystones to increase the compliance for office hysteroscopy. Furthermore, we have demonstrated in a PRCT that by reducing the instrument diameter, both, surgeons’ experience and cervical canal passage did not interfere in the overall success rate of this technique. The value of this concept was recently confirmed in the Trophy study published in the Lancet 2016. (El-Thoukhy et al., 2016).

Hysteroscopy has come a long way up to the combination of ultrasound, Campo Trophyscope ® hysteroscopy, contrast sonography and mini surgical instruments providing the possibility for every trained examiner to perform a one stop uterine diagnosis, including endo or myometrial tissue sampling.

An up-coming new era of major technical improvements in the manufacturing of high-quality small-bored rigid optics, including the 15 Fr. office resectoscope, the 19 Fr. Intrauterine BIGATTI Shaver (IBS ® ) and the Campo Trophyscope ® hysteroscope is expected. This has given a perfect answer to the challenges of an ambulatory hysteroscopic surgical unit performing interventions under local or conscious sedation, resulting in optimized total procedure time, increased safety, patient satisfaction and accessibility for up to 90 % of all hysteroscopic interventions.

The knowledge of outpatient hysteroscopy potential, but also its limits for a clinical practice, is the starting point for the diffusion of the technique and the enlargement of its field of application.
